# *BRCA1* Promoter Methylation Marks a Shared Blood Methylome State in Women with and Without Breast Cancer

**DOI:** 10.3390/ijms27209289

**Published:** 2026-10-19

**Authors:** Karolina Prajzendanc, Katarzyna Ewa Sokołowska, Tomasz Kazimierz Wojdacz, Jan Lubiński, Jacek Gronwald, Anna Jakubowska

**Affiliations:** 1 Department of Genetics and Pathology, International Hereditary Cancer Center, Pomeranian Medical University, 70-204 Szczecin, Poland; 2 Regional Center for Digital Medicine, Pomeranian Medical University, 70-204 Szczecin, Poland; 3 The Independent Clinical Epigenetics Laboratory, Pomeranian Medical University, 70-204 Szczecin, Poland; 4 Independent Laboratory of Molecular Biology and Genetic Diagnostics, Pomeranian Medical University, 70-204 Szczecin, Poland

**Keywords:** cancer epigenetics, DNA methylation, cancer biomarker

## Abstract

Aberrant DNA methylation in peripheral blood has been proposed as a marker of breast cancer (BC) susceptibility. To determine whether *BRCA1* promoter methylation reflects a broader constitutional methylation state, we performed genome-wide DNA methylation profiling in peripheral blood from 24 women with triple-negative breast cancer (TNBC) and 35 healthy controls, all carrying *BRCA1* promoter methylation. Analysis using the Infinium MethylationEPIC BeadChip revealed no significant differences in global methylation patterns between groups. These findings suggest that *BRCA1* promoter methylation represents a shared epigenetic state established before disease onset and largely unaffected by subsequent breast cancer development.

## 1. Introduction

DNA methylation patterns in peripheral blood are increasingly recognized as stable epigenetic features that may reflect early-established biological states which, when aberrant, may be associated with disease susceptibility [1,2]. In the context of breast cancer (BC), aberrant DNA methylation detected in blood has been proposed as a marker of cancer risk, potentially originating during early development or embryogenesis and persisting throughout life [3]. Among such alterations, methylation of the *BRCA1* gene promoter has been repeatedly associated with increased risk of breast cancer, particularly triple-negative breast cancer (TNBC), and is detectable in peripheral blood long before diagnosis [4,5,6]. However, it remains unclear whether *BRCA1* promoter methylation represents an isolated epigenetic event or instead marks a broader, coordinated alteration of the blood methylome that predisposes to disease.

Previous genome-wide DNA methylation studies in BC have predominantly compared patients and healthy controls without stratification for specific epigenetic alterations present in peripheral blood [7,8,9,10]. Such studies revealed various number of differentially methylated positions (DMPs) between cases and controls [11]. Whether these signals reflect effects of cancer itself or underlying epigenetic heterogeneity related to cancer susceptibility remains unresolved. While some DNA methylation patterns are established in utero, a wide range of environmental factors can modify the methylome [12]. Several studies have reported greater DNA methylation differences between cases and controls when the time to diagnosis was shorter, suggesting that the development of BC may act as such a modifying factor [13,14]. However, focusing specifically on *BRCA1* methylation, it remains unknown whether individuals sharing *BRCA1* promoter methylation also share a broader blood methylome state and whether BC onset introduces additional, disease-specific methylation changes beyond this pre-existing epigenetic background.

Here, we performed genome-wide DNA methylation profiling of peripheral blood from women with and without breast cancer, all exhibiting *BRCA1* promoter methylation. By restricting the analysis to individuals sharing this epigenetic marker, we aimed to test whether BC status is associated with additional global blood methylome alterations. We hypothesized that if *BRCA1* promoter methylation marks a broader, early-established epigenetic state, genome-wide methylation profiles would be similar between patients and controls.

## 2. Results

Comparison of genome-wide DNA methylation profiles between TNBC patients and healthy controls with *BRCA1* promoter methylation revealed eight differentially methylated CpG sites located in six genes (*PIWIL1*, *CIT*, *SLC37A1*, *COL1A2*, *TNFRSF6B*, *GPR1-AS*) and one intergenic region, however none of these were statistically significant. Details of identified CpGs are presented in Table 1.

To further investigate global methylation patterns and evaluate potential group stratification, a Principal Component Analysis (PCA) was performed using the 10,000 most variable CpG sites across all samples. The PCA plot showed no distinct clustering or separation between TNBC cases and healthy controls based on their breast cancer status (Supplementary Figure S1).

## 3. Discussion

Although DNA methylation has been studied for decades, the mechanisms underlying the establishment and maintenance of specific methylation patterns remain incompletely understood. Accumulating evidence suggests that both genetic background and stochastic or environmental influences acting early in life may contribute to coordinated methylation changes across multiple genomic regions and tissues [15,16]. In this context, constitutional promoter methylation of *BRCA1* represents a well-characterized example of an early-established epigenetic alteration, detectable in peripheral blood, and associated with increased BC risk [17].

The primary aim of this study was to determine whether BC status is associated with global blood methylome alterations in individuals sharing *BRCA1* promoter methylation. We hypothesized that if *BRCA1* promoter methylation reflects a broader, early-established epigenetic state, genome-wide blood DNA methylation profiles would be largely similar between BC patients and healthy controls. Genome-wide methylation analysis of 24 TNBC cases and 35 healthy controls revealed no statistically significant differences in mean methylation levels between both groups. Additionally, PCA of 10,000 most variable CpGs demonstrated that TNBC patients and controls do not form distinct clusters based on their clinical status. These findings indicate that the global blood methylome seems to be largely conserved in affected and unaffected individuals, supporting the interpretation of *BRCA1* promoter methylation as a marker of a shared epigenetic state independent of disease status. However, healthy individuals still have an increased risk of developing BC in the future due to the presence of *BRCA1* promoter methylation and, potentially, an accompanying genome-wide methylation pattern [18].

To further understand the molecular nuances of this shared epigenetic background, our genome-wide analysis highlighted eight CpG sites with the largest effect sizes (Delta β ≥ 0.1, Hedges’ g ≥ 0.8), located within six genes (*PIWIL1*, *CIT*, *SLC37A1*, *COL1A2*, *TNFRSF6B*, *GPR1-AS*) and one intergenic region. While these sites did not reach strict genome-wide statistical significance after FDR correction, and must therefore be interpreted strictly as exploratory and hypothesis-generating, their specific molecular functions may provide intriguing insights into the biological landscape associated with this constitutional susceptibility.

Specifically, two DMPs were located in the promoter region of the *PIWIL1* gene, up to 200 nucleotides before the transcription start site. In TNBC patients, the mean methylation level was lower than in controls. Although these two CpGs are located within a dense CpG island, this localized hypomethylation nominally aligns with the well-documented overexpression of *PIWIL1* in breast and other cancers [19,20,21]. The Piwil1 protein plays a fundamental role in the piRNA pathway, driving the silencing of transposable elements and participating in stem cell self-renewal. Interestingly, Piwil1 promotes DNA methylation via the upregulation of DNA methyltransferase 1 (DNMT1) [22]. In the context of our study, rather than reflecting a tumor-induced alteration, this directional trend towards lower methylation at the *PIWIL1* promoter in TNBC patients could speculatively illuminate potential intrinsic molecular heterogeneity within this shared susceptibility background [23].

A similar pattern of potential molecular alterations can be inferred from the *TNFRSF6B* gene, which encodes Decoy receptor 3 (DcR3)—a protein that neutralizes cytotoxic ligands and protects against apoptosis [24]. We detected a hypermethylated CpG site within the 3′UTR region and first exon of *TNFRSF6B* in TNBC patients. Given that gene body methylation is frequently associated with active transcription and increased gene expression, the observed hypermethylation nominally aligns with the known oncogenic role of DcR3 in promoting breast cancer cell migration and invasion [25,26]. Furthermore, we observed hypermethylation within the gene body of *CIT* (Citron kinase), a critical regulator of cytokinesis and mitosis that promotes aggressiveness and tumorigenesis in breast cancer cells [27]. In our analysis, the CpG located in the *CIT* gene body was hypermethylated in BC patients, speculatively pointing toward an elevated expression.

The single CpG located in exon 19 of *COL1A2* (involved in extracellular matrix organization) was hypermethylated in breast cancer patients, suggesting a potential upregulation [28]. Similarly, a hypermethylated CpG was found in intron 18 of *SLC37A1*, a gene upregulated in breast cancer cells by EGF. While the impact of intronic methylation remains complex, it may critically affect alternative splicing [29,30,31]. Lastly, the single CpG in the first intron of the antisense RNA *GPR1-AS* was hypomethylated in patients, and another hypomethylated CpG was located in an enhancer region on chromosome 6 regulating *ZNF391*. Together, these alterations in non-coding and regulatory elements may point to a coordinated baseline drift that may modulate the transcriptional landscape of peripheral blood cells. Ultimately, these findings cannot support definitive mechanistic conclusions but serve as valuable candidate loci for future functional and validation studies.

This pronounced consistency in the global blood methylome—interrupted only by these subtle shifts in key regulatory loci—points toward a robust somatic stability maintained by the core epigenetic machinery [32]. The fundamental maintenance of DNA methylation patterns is likely governed by a highly synchronized activity of DNA methyltransferases (such as DNMT1, DNMT3A, and DNMT3B) and ten-eleven translocation (TET) dioxygenases. Crucially, BRCA1 protein has been established as a direct positive regulator of *DNMT1* expression, and its deficiency is known to prompt global DNA hypomethylation and genomic instability in target tissues [32,33]. However, DNA methylation marks in hematopoietic lineages are characterized by a robust epigenetic memory that preserves lineage-specific stability throughout differentiation [34]. In our study, no FDR-significant differences or large methylation changes were identified in the peripheral blood methylome between healthy controls and TNBC patients. Consequently, while modest epigenetic effects cannot be ruled out, these findings suggest that constitutional *BRCA1* methylation may establish a shared, compensated epigenetic baseline prior to disease onset. Within the blood compartment, any potential defect in DNMT1 maintenance driven by *BRCA1* silencing may be effectively counterbalanced by the homeostatic interplay of de novo methyltransferases and TET enzymes, or expression of DNMT1 may be offset by Piwil1 upregulation, preserving global epigenetic balance [22]. Our findings suggest that once this specific epigenetic landscape is established during early development, the underlying molecular machinery appears to remain resistant to systemic perturbations [35].

Importantly, because blood samples from patients were collected at the time of diagnosis rather than years before, our results indicate that the development of BC did not measurably affect blood methylome within the timeline captured by our study. However, this observation contrasts with several previous studies reporting that the time interval between blood draw and BC diagnosis correlates with the magnitude of observed methylation differences between cases and controls [8,11,36]. It has been suggested that at least some genome-wide methylation changes detected in patients may arise as a consequence of developing cancer and could therefore serve as predictive biomarkers of short-term BC risk [14]. However, these two observations, methylation changes predisposing to cancer and alterations induced by cancer, may proceed in parallel and interact, thereby influencing both cancer development and the methylome state [37,38]. Overall, our findings support the hypothesis that women with *BRCA1* promoter methylation display a specific and potentially conserved baseline methylome profile that appears relatively stable against modification by internal or external factors associated with early-stage disease [33,35]. This specific methylome seems to be markedly different from that observed in women without detectable *BRCA1* promoter methylation, independent of BC status [39].

Taken together, these findings support a model in which blood-based DNA methylation alterations associated with BC risk may arise early in life and remain largely stable thereafter, rather than being dynamically reshaped by disease onset. However, this stability may be restricted to specific methylome patterns that, for reasons not yet fully understood, are more resistant to modification by environmental factors. In this framework, *BRCA1* promoter methylation does not represent an isolated epigenetic lesion but rather serves as a marker of a broader, coordinated methylome state established prior to cancer development. The lack of genome-wide methylation differences between cases and controls suggests that once this epigenetic background is present, subsequent BC development does not substantially modify the blood methylome, although modest epigenetic effects, and transient or stage-specific perturbations cannot be entirely ruled out.

While these conceptual frameworks offer a compelling explanation for our findings, they also underscore the need for a cautious interpretation. Several limitations of this study should be acknowledged. First, the sample size was relatively modest, which limits the power to detect subtle DNA methylation differences between groups. Second, the cross-sectional design of our study, where blood was sampled at a single timepoint (at diagnosis), prevents us from tracking potential dynamic or treatment-induced epigenetic fluctuations over time. Third, the analysis was restricted to individuals with *BRCA1* promoter methylation as a stratifying marker. Future studies incorporating additional epigenetic features and larger, independent cohorts will be required to further validate the permanence of this shared epigenetic baseline. Additionally, extending this analysis to include a *BRCA1*-unmethylated reference group would be highly valuable to fully delineate the specific boundaries of this profile.

In conclusion, our genome-wide analysis demonstrates that women sharing *BRCA1* promoter methylation exhibit similar blood DNA methylation profiles at the time of blood sampling, with no statistically significant differences detected between cases and controls. These findings support the interpretation of *BRCA1* promoter methylation as a marker of a broader, early-established epigenetic state that probably remains stable at the time of cancer diagnosis. Incorporating epigenetic stratification into blood-based methylation studies may therefore be essential for accurately distinguishing susceptibility-related epigenetic variation from changes attributable to cancer itself.

## 4. Material and Methods

From our previously described cohort [4], we selected 24 women with TNBC and 35 healthy controls for whom high-quality DNA samples were available. In all cases and controls, *BRCA1* promoter methylation in peripheral blood DNA had been detected using methylation-sensitive high-resolution melting (MS-HRM) analysis [40]. The mean year of birth was 1952 (range: 1929–1981) for cases and 1953 (range: 1923–1977) for controls. The mean age at diagnosis was 56 years (range: 31–78). The mean age at sample collection was 60 years (range: 37–90 years) in the control group. Blood samples from patients were collected at the time of diagnosis. All cases and controls were of Polish ethnicity and found to be negative for the 13 recurrent Polish germline *BRCA1/2* pathogenic variants. All patients were selected from the registry of International Hereditary Cancer Center Department of Genetics and Pathology Pomeranian Medical University in Szczecin.

Genome-wide DNA methylation profiling was performed using the Infinium MethylationEPIC BeadChip array (Illumina, San Diego, CA, USA), interrogating over 850,000 CpG sites across the genome. Arrays were scanned using the iScan System (Illumina, San Diego, CA, USA). Raw DNA methylation data were processed using the ChAMP R package (R software, version 4.5.1). Following data preprocessing, DNA methylation levels were obtained for 738 403 CpG sites. Genome-wide analysis was performed using the same DNA samples that had previously been used for *BRCA1* methylation analysis by MS-HRM.

Methylation profiling data were corrected for cell fraction (Supplementary Tables S1–S3) proportions using outperforming robust partial correlation method implemented in EpiDISH R package (R software, version 4.5.1) with “centDHSbloodDMC.m” dataset as reference [41,42]. We defined deferentially methylation CpG sites as these with DNA methylation difference (delta β-value) between cases and controls equal or greater than 0.1 and displayed the Hedges’ g effect size of at least 0.8, which is the measure of standardized by variance difference between means [43]. We used linear regression method implemented in ChAMP R package to investigate if differentially methylated CpGs are statistically significant (FDR-corrected *p*-value < 0.05).

## Figures and Tables

**Table 1:** CpGs with highest delta β-value (≥0.1) and the Hedges’ g effect ≥0.8.

CpG	Chr.	Position	Gene	Controls Mean Meth.	Patients Mean Meth.	Delta β	SD	Var	Adj. *p*-Value
cg04192258	21	43999785	*SLC37A1*	0.62	0.74	0.12	0.11	0.08	0.16
cg17338594	12	120173114	*CIT*	0.72	0.86	0.13	0.12	0.08	0.16
cg11931211	12	130822361	*PIWIL1*	0.67	0.54	−0.13	0.14	0.08	0.36
cg27630820	12	130822294	*PIWIL1*	0.72	0.6	−0.13	0.13	0.08	0.34
cg27508180	7	94039079	*COL1A2*	0.75	0.86	0.1	0.09	0.08	0.16
cg22444562	2	207090465	*GPR1-AS*	0.52	0.29	−0.23	0.27	0.07	0.17
cg16702083	20	62328427	*TNFRSF6B*	0.27	0.38	0.12	0.13	0.07	0.31
cg06168204	6	27570548	-	0.46	0.36	−0.11	0.12	0.07	0.19

SD—standard deviation; var—variance.

## Data Availability

The raw data supporting the conclusions of this article will be made available by the authors on request.

## Supplementary Materials

The following supporting information can be downloaded at: https://www.mdpi.com/article/10.3390/ijms27209289/s1.

## Abbreviations

BC	breast cancer
DMP	differentially methylated position
DNMT	DNA methyltransferase
MS-HRM	methylation-sensitive high-resolution melting
PCA	principal component analysis
SD	standard deviation
TET	ten-eleven translocation
TNBC	triple-negative breast cancer

## References

[1] Dor Y., Cedar H. (2018). Principles of DNA methylation and their implications for biology and medicine. Lancet.

[2] Wu H.-C., John E.M., Ferris J.S., Keegan T.H., Chung W.K., Andrulis I., Delgado-Cruzata L., Kappil M., Gonzalez K., Santella R.M. (2011). Global DNA methylation levels in girls with and without a family history of breast cancer. Epigenetics.

[3] La Cruz M.R.-D., MartínEz-Gregorio H., DíaZ-VelásQuez C.E., Ambriz-Barrera F., Resendiz-Flores N.G., Gitler-Weingarten R., Rojo-Castillo M.P., Pradda D., Oliver J., Perdomo S. (2024). Methylation marks in blood DNA reveal breast cancer risk in patients fulfilling hereditary disease criteria. npj Precis. Oncol..

[4] Prajzendanc K., Domagała P., Hybiak J., Ryś J., Huzarski T., Szwiec M., Tomiczek-Szwiec J., Redelbach W., Sejda A., Gronwald J. (2019). *BRCA1* promoter methylation in peripheral blood is associated with the risk of triple-negative breast cancer. Int. J. Cancer.

[5] Wong E.M., Southey M.C., Fox S.B., Brown M.A., Dowty J.G., Jenkins M.A., Giles G.G., Hopper J.L., Dobrovic A. (2011). Constitutional Methylation of the *BRCA1* Promoter Is Specifically Associated with *BRCA1* Mutation-Associated Pathology in Early-Onset Breast Cancer. Cancer Prev. Res..

[6] Lønning P.E., Knappskog S. (2018). BRCA1 methylation in newborns: Genetic disposition, maternal transfer, environmental influence, or by chance only?. Clin. Epigenet..

[7] Joo J.E., Dowty J.G., Milne R.L., Wong E.M., Dugué P.-A., English D., Hopper J.L., Goldgar D.E., Giles G.G., Southey M.C. (2018). Heritable DNA methylation marks associated with susceptibility to breast cancer. Nat. Commun..

[8] Xu Z., Bolick S.C., DeRoo L.A., Weinberg C.R., Sandler D.P., Taylor J.A. (2013). Epigenome-wide Association Study of Breast Cancer Using Prospectively Collected Sister Study Samples. JNCI J. Natl. Cancer Inst..

[9] Polidoro S., Johansson H., Cugliari G., Guerrieri-Gonzaga A., Aristarco V., Macis D., Calvello M., Marabelli M., Feroce I., Serrano D. (2025). Impact of Epigenome-Wide Methylation and Breast Cancer Recurrence in Women Tested Negative for BRCA Genes: The Breast Methylation Risk (BREMERI) Study. Cancers.

[10] Tang Q., Holland-Letz T., Slynko A., Cuk K., Marme F., Schott S., Heil J., Qu B., Golatta M., Bewerunge-Hudler M. (2016). DNA methylation array analysis identifies breast cancer associated *RPTOR*, *MGRN1* and *RAPSN* hypomethylation in peripheral blood DNA. Oncotarget.

[11] Ennour-Idrissi K., Dragic D., Durocher F., Diorio C. (2020). Epigenome-wide DNA methylation and risk of breast cancer: A systematic review. BMC Cancer.

[12] Li S., Wong E.M., Dugué P.-A., McRae A.F., Kim E., Joo J.-H.E., Nguyen T.L., Stone J., Dite G.S., Armstrong N.J. (2018). Genome-wide average DNA methylation is determined in utero. Leuk. Res..

[13] Severi G., Southey M.C., English D.R., Jung C.-H., Lonie A., McLean C., Tsimiklis H., Hopper J.L., Giles G.G., Baglietto L. (2014). Epigenome-wide methylation in DNA from peripheral blood as a marker of risk for breast cancer. Breast Cancer Res. Treat..

[14] Xu Z., Sandler D.P., A Taylor J. (2019). Blood DNA Methylation and Breast Cancer: A Prospective Case-Cohort Analysis in the Sister Study. JNCI J. Natl. Cancer Inst..

[15] Bergman Y., Cedar H. (2013). DNA methylation dynamics in health and disease. Nat. Struct. Mol. Biol..

[16] Vaidya H., Jelinek J., Issa J.-P.J. (2025). DNA Methylation, Aging, and Cancer. Epigenomes.

[17] Lønning P.E., Nikolaienko O., Pan K., Kurian A.W., Eikesdal H.P., Pettinger M., Anderson G.L., Prentice R.L., Chlebowski R.T., Knappskog S. (2022). Constitutional *BRCA1* Methylation and Risk of Incident Triple-Negative Breast Cancer and High-grade Serous Ovarian Cancer. JAMA Oncol..

[18] Nikolaienko O., Eikesdal H.P., Ognedal E., Gilje B., Lundgren S., Blix E.S., Espelid H., Geisler J., Geisler S., Janssen E.A.M. (2023). Prenatal BRCA1 epimutations contribute significantly to triple-negative breast cancer development. Genome Med..

[19] Litwin M., Szczepańska-Buda A., Michałowska D., Grzegrzółka J., Piotrowska A., Gomułkiewicz A., Wojnar A., Dzięgiel P., Witkiewicz W. (2018). Aberrant Expression of PIWIL1 and PIWIL2 and Their Clinical Significance in Ductal Breast Carcinoma. Anticancer. Res..

[20] Wang Y., Liu Y., Shen X., Zhang X., Chen X., Yang C., Gao H. (2012). The PIWI protein acts as a predictive marker for human gastric cancer. Int. J. Clin. Exp. Pathol..

[21] Sun R., Gao C.-L., Li D.-H., Li B.-J., Ding Y.-H. (2017). Expression Status of PIWIL1 as a Prognostic Marker of Colorectal Cancer. Dis. Markers.

[22] Siddiqi S., Matushansky I. (2012). Piwis and piwi-interacting RNAs in the epigenetics of cancer. J. Cell. Biochem..

[23] Teschendorff A.E., Jones A., Widschwendter M. (2016). Stochastic epigenetic outliers can define field defects in cancer. BMC Bioinform..

[24] Wu N.-L., Huang D.-Y., Hsieh S.-L., Dai Y.-S., Lin W.-W. (2022). Decoy receptor 3 is involved in epidermal keratinocyte commitment to terminal differentiation via EGFR and PKC activation. Exp. Mol. Med..

[25] Hsieh S.-L., Lin W.-W. (2017). Decoy receptor 3: An endogenous immunomodulator in cancer growth and inflammatory reactions. J. Biomed. Sci..

[26] Ge Z., Sanders A.J., Ye L., Wang Y., Jiang W.G. (2011). Expression of death decoy receptor-3 (DcR3) in human breast cancer and its functional effects on breast cancer cells in vitro. J. Exp. Ther. Oncol..

[27] Meng D., Yu Q., Feng L., Luo M., Shao S., Huang S., Wang G., Jing X., Tong Z., Zhao X. (2018). Citron kinase (CIT-K) promotes aggressiveness and tumorigenesis of breast cancer cells in vitro and in vivo: Preliminary study of the underlying mechanism. Clin. Transl. Oncol..

[28] Hwang S.J., Ha G.-H., Seo W.-Y., Kim C.K., Kim K., Lee S.B. (2020). Human collagen alpha-2 type I stimulates collagen synthesis, wound healing, and elastin production in normal human dermal fibroblasts (HDFs). BMB Rep..

[29] Iacopetta D., Lappano R., Cappello A.R., Madeo M., De Francesco E.M., Santoro A., Curcio R., Capobianco L., Pezzi V., Maggiolini M. (2009). SLC37A1 Gene expression is up-regulated by epidermal growth factor in breast cancer cells. Breast Cancer Res. Treat..

[30] Maor G.L., Yearim A., Ast G. (2015). The alternative role of DNA methylation in splicing regulation. Trends Genet..

[31] Wong J.J.-L., Gao D., Nguyen T.V., Kwok C.-T., van Geldermalsen M., Middleton R., Pinello N., Thoeng A., Nagarajah R., Holst J. (2017). Intron retention is regulated by altered MeCP2-mediated splicing factor recruitment. Nat. Commun..

[32] Moore L.D., Le T., Fan G. (2013). DNA Methylation and Its Basic Function. Neuropsychopharmacology.

[33] Shukla V., Coumoul X., Lahusen T., Wang R.-H., Xu X., Vassilopoulos A., Xiao C., Lee M.-H., Man Y.-G., Ouchi M. (2010). BRCA1 affects global DNA methylation through regulation of DNMT1. Cell Res..

[34] Blecua P., Martinez-Verbo L., Esteller M. (2020). The DNA methylation landscape of hematological malignancies: An update. Mol. Oncol..

[35] Danielewski M., Walkowiak J., Wielgus K., Nowak J.K. (2024). Stability of Blood DNA Methylation Across Two Timepoints in Three Cohorts. Biomedicines.

[36] van Veldhoven K., Polidoro S., Baglietto L., Severi G., Sacerdote C., Panico S., Mattiello A., Palli D., Masala G., Krogh V. (2015). Epigenome-wide association study reveals decreased average methylation levels years before breast cancer diagnosis. Clin. Epigenet..

[37] Esteller M. (2008). Epigenetics in cancer. N. Engl. J. Med..

[38] Besselink N., Keijer J., Vermeulen C., Boymans S., de Ridder J., van Hoeck A., Cuppen E., Kuijk E. (2023). The genome-wide mutational consequences of DNA hypomethylation. Sci. Rep..

[39] KE S., Antoniewski J., Sobalska-Kwapis M., Strapagiel D., Lubiński J., Huzarski T., Wojdacz T.K. (2025). Blood cells of BRCA1 mutation and epimutation carries appear to acquire specific epigenetic signatures. Research Square.

[40] Wojdacz T.K., Dobrovic A., Hansen L.L. (2008). Methylation-sensitive high-resolution melting. Nat. Protoc..

[41] Teschendorff A.E., Breeze C.E., Zheng S.C., Beck S. (2017). A comparison of reference-based algorithms for correcting cell-type heterogeneity in Epigenome-Wide Association Studies. BMC Bioinform..

[42] Houseman E.A., Accomando W.P., Koestler D.C., Christensen B.C., Marsit C.J., Nelson H.H., Wiencke J.K., Kelsey K.T. (2012). DNA methylation arrays as surrogate measures of cell mixture distribution. BMC Bioinform..

[43] Sullivan G.M., Feinn R. (2012). Using Effect Size—Or Why the P Value Is Not Enough. J. Grad. Med. Educ..

